# Affective Priming by Eye Gaze Stimuli: Behavioral and Electrophysiological Evidence

**DOI:** 10.3389/fnhum.2016.00619

**Published:** 2016-12-07

**Authors:** Tingji Chen, Mikko J. Peltola, Lotta J. Ranta, Jari K. Hietanen

**Affiliations:** Human Information Processing Laboratory, School of Social Sciences and Humanities/Psychology, University of TampereTampere, Finland

**Keywords:** eye gaze, affective priming, event-related potential, self-ratings, affective evaluation

## Abstract

The present study employed the affective priming paradigm and measurements of event-related potentials (ERPs) to investigate implicit affective reactions elicited by gaze stimuli. Participants categorized positive and negative words primed by direct gaze, averted gaze and closed eyes. The behavioral response time (RT) results indicated that direct gaze implicitly elicited more positive affective reactions than did closed eyes. Analyses of the ERP responses to the target words revealed a priming effect on the N170 and an interaction on late positive potential (LPP) responses, and congruently with the behavioral results, suggested that, compared to closed eyes, direct gaze was affectively more congruent with positive words and more incongruent with negative words. The priming effect on the N170 response indicated that gaze stimuli influenced the subsequent affective word processing at an early stage of information processing. In conclusion, the present behavioral and electrophysiological evidence suggests that direct gaze automatically activates more positive affective reactions than closed eyes.

## Introduction

Gaze and eye contact are crucial social signals, which provide information about individuals’ direction of attention and intentions. A gaze looking towards the perceiver is likely to be interpreted as signaling a tendency to approach and intention to communicate, whereas eyes looking away may signal a tendency to avoid (Argyle and Cook, 1976). Eye gaze not only signals the sender’s approach-avoidance tendencies, but it also activates corresponding motivational tendencies in the perceivers. Seeing another person’s direct gaze, as compared to averted gaze or closed eyes, elicits greater relative left-side frontal Electroencephalographic (EEG) activity, associated with approach tendency (Harmon-Jones, 2003; Hietanen et al., 2008; Pönkänen et al., 2011b; Kylliäinen et al., 2012). Studies investigating the skin conductance response (SCR), a measure of physiological arousal, have shown greater SCRs in response to direct gaze as compared to averted gaze or closed eyes (Nichols and Champness, 1971; Hietanen et al., 2008; Helminen et al., 2011). These findings indicate that eye gaze is a powerful stimulus eliciting affective-motivational responses in the perceiver.

In addition to eliciting physiological arousal and motivational responses, eye gaze has also been shown to elicit affective responses in the perceiver. However, studies measuring explicit responses, such as self-rated affective valence, have indicated somewhat contradicting findings of both negative and positive affective responses to another individual’s direct gaze. Direct gaze can signal dominance and aggression, thus eliciting aversive responses in both animals and humans (Emery, 2000; Skuse, 2003). Accordingly, studies investigating self-reported affective responses to eye gaze have revealed that, for emotionally neutral faces, direct gaze is evaluated as less positive than averted gaze and closed eyes (Hess et al., 2007; Hietanen et al., 2008; Pönkänen et al., 2011a). On the other hand, direct gaze can be perceived as a signal of positive communicative intention (Kleinke, 1986). Studies investigating self-reported likability and attractiveness of faces have shown that participants evaluate faces with direct gaze as more likable and attractive than faces with averted gaze (Mason et al., 2005; Kuzmanovic et al., 2009; Ewing et al., 2010). Some studies using physiological measurements or indirect behavioral measurements, suggest the evaluation of direct gaze in a more positive manner (Kampe et al., 2001; Lawson, 2015). For example, using the Implicit Association Test, Lawson (2015) showed a robust preference for faces looking towards the perceiver, than looking away. Thus, it can be speculated that the nature of affective responses to gaze direction may depend on whether explicit or implicit affective reactions are measured. As compared with implicit responses, explicit responses are more susceptible to task demands, motivational biases, and other top-down influences (Hofmann et al., 2005). By contrast, implicit responses, which do not rely on introspective experience, may be less susceptible to motivational influence, and may reflect perceivers’ instinctual response to a direct gaze.

In support of this speculation, we recently showed that implicit and explicit measures, indeed, resulted in differential affective evaluations (Chen et al., 2016). We used the affective priming paradigm (Fazio et al., 1986) to investigate automatic (implicit) affective responses elicited by eye gaze. In a typical affective priming experiment, participants are required to make speeded evaluations of affective targets preceded by briefly presented affective primes. Typically, the response latency to targets is shorter for the affectively congruent prime-target pairs compared to affectively incongruent pairs (Fazio et al., 1986). This finding has been interpreted to indicate that the presentation of the prime automatically elicits the associated affective evaluation and facilitates the decoding of affectively congruent targets (Fazio et al., 1986; Fazio, 2001). Since the seminal work by Fazio et al. (1986), the affective priming effect has been reported in a large number of studies and demonstrated to be a replicable and robust phenomenon (Fazio, 2001; Klauer and Musch, 2003). In our previous affective priming study, the gaze stimuli were briefly presented as primes and immediately followed by positive and negative words as targets. The participants evaluated the valence of the words as fast as possible. Responses to positive words were faster after direct gaze than after closed eyes, while responses to negative words were faster after closed eyes than after direct gaze. These results were interpreted to indicate that the perception of direct gaze automatically activates more positive evaluations than closed eyes (Chen et al., 2016). Instead, the explicit ratings showed that direct gaze was evaluated as less positive than closed eyes.

However, with the behavioral response time (RT) data alone, it is impossible to know at which stage of processing gaze primes start to exert their influence on target processing. The high temporal resolution of event-related potentials (ERPs), which provide a continuous time-window on neural processes during stimulus presentation, may shed light on this question. Importantly, previous studies have demonstrated that some ERP components are modulated by the affective congruence between targets and preceding contexts. Investigation of these components may provide indications about the affective (in)congruence between gaze stimuli and affective words on the neural level.

In the present study, we employed the affective priming paradigm (as in Chen et al., 2016) and recorded ERPs in response to the target words preceded by eye gaze. Previous studies employing a similar methodology have suggested that P1 is the earliest component to be modulated in affective priming studies, with larger P1 amplitudes in response to affectively incongruent vs. congruent targets (Hietanen and Astikainen, 2013; Sianipar et al., 2015). The following N170 component, with time window around 150–200 ms after target onset (earlier for face and later for word targets), has also been reported to be modulated in affective priming studies with words (Comesaña et al., 2013) and faces (Hietanen and Astikainen, 2013; Hinojosa et al., 2015) as affective targets. For example, Hietanen and Astikainen (2013) showed that happy faces preceded by pictures of positive emotional scenes elicited larger N170 amplitudes, whereas the N170 amplitudes were larger for sad faces preceded by negative scenes. The N170 component reflects early visual processing of faces, words, and objects (Rossion et al., 2000). In the context of affective priming, modulation of the N170 response by affective primes may indicate increased activity in visual processing areas when the affective contents of the prime and target match. A few studies have shown priming effects on the early posterior negativity (EPN), with enhanced EPN amplitudes (i.e., more negative mean activity at around 250–400 ms), in response to incongruent vs. congruent targets (Hietanen and Astikainen, 2013; Rampone et al., 2014). The midline N400 component is suggested to reflect semantic integration of words with the preceding context (Kutas and Hillyard, 1980) and it has been reported to be modulated by the affective congruence between words and the preceding context (Zhang et al., 2006, 2010; Eder et al., 2011). Typically, greater N400 amplitudes are observed for affectively incongruent than for congruent pairs. Late positive potential (LPP) modulation by affective congruence has been reported in priming studies with words (Zhang et al., 2010), faces (Werheid et al., 2005; Hietanen and Astikainen, 2013), and scenes (Herring et al., 2011) as affective targets. The results have revealed greater LPP amplitudes at around 400–700 ms to affectively incongruent than congruent targets. EPN and LPP are both suggested to reflect selective attention to emotionally significant stimuli (Schupp et al., 2004).

In the present study, we aimed to: (i) replicate our previous results of implicit and explicit affective responses to eye gaze; (ii) provide physiological evidence related to affective congruence of gaze-word pairs by investigating those ERP components which have been demonstrated to be modulated by the affective congruence between targets and preceding primes; and (iii) by measuring ERP responses, examine the earliest stage of processing at which gaze stimuli start to exert their influence on the processing of affective words. We expected to replicate the behavioral results of our previous study (Chen et al., 2016). Namely, in the affective priming task, positive words would be responded to faster after direct gaze than after closed eyes, while negative words would be responded to faster after closed eyes than after direct gaze (i.e., positive words being affectively more congruent with direct gaze than closed eyes). In the self-rating task, on the other hand, direct gaze would be evaluated as less positive compared to closed eyes. Based on the previous ERP studies (Zhang et al., 2010; Herring et al., 2011; Hietanen and Astikainen, 2013), we expected affective priming effects on the P1, N170, EPN, N400 and LPP components. To be specific, P1, EPN, N400 and LPP responses to positive words were expected to be larger when preceded by closed eyes vs. direct gaze, whereas these responses to negative words would be larger when preceded by direct gaze vs. closed eyes. The N170 response to positive words was expected to be greater when preceded by direct gaze vs. closed eyes, whereas N170 to negative words would be greater when preceded by closed eyes vs. direct gaze.

## Materials and Methods

### Participants

Thirty-two native Finnish speakers (17 females; 19–32 years, mean = 25 years), with normal or corrected-to-normal vision, participated. Three participants were excluded from the ERP data analyses due to technical errors. All participants were informed about the experimental procedure, and a signed consent was obtained from each participant. The participants were given a movie ticket for their participation. The research protocol was approved by the Ethics Committee of the Tampere region.

### Stimuli

**Figure 1:**
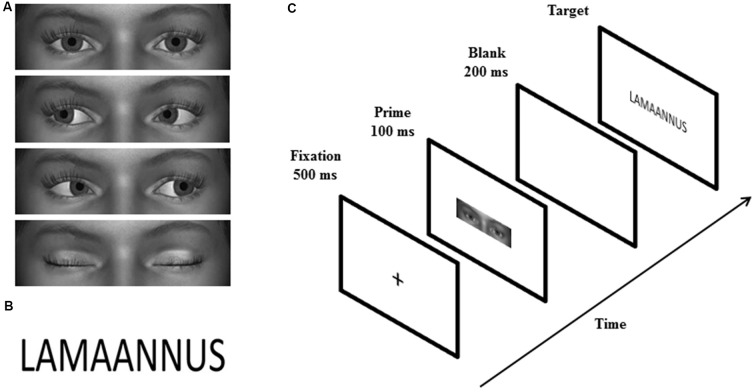
**Examples of the prime (A) and target (B) stimuli. The sequence of events on a single trial is illustrated in (C)**.

Similar to the study by Chen et al. (2016), the primes were grayscale images showing a rectangular shaped area of the eye region of animated faces. Four male and four female faces with direct gaze, averted gaze (20° left/right), and closed eyes (Figure 1A) were created by using a 3D animation software, Digital Art Zone [Daz] 3D Studio^1^. The size of the primes was 2.0° × 6.9° vertically and horizontally, respectively. The targets were 48 positive and 48 negative Finnish words selected from the study by Söderholm et al. (2013) (Figure 1B). According to the ratings made by 20–30-year-old participants (Söderholm et al., 2013), positive and negative words differed significantly in valence ratings, *t*_(94)_ = 34.56, *p* < 0.001 (*M*_positive_ = 5.62; *M*_negative_ = 2.55), but not in arousal (*p* = 0.579), word length (*p* = 0.691), or absolute or relative surface frequency (both *p*s = 0.787). The targets were displayed in Calibri font and the size of the words presented on the screen was 1.5° vertically and 3.8°–6.9° horizontally.

### Design and Procedure

The experiment consisted of a priming task and an explicit rating task. The priming task had a 3 (gaze: direct, averted, closed) × 2 (word valence: positive vs. negative) design, including six blocks with 96 trials in each. Each of the 96 targets (48 positive and 48 negative words) were paired twice with a direct gaze, averted gaze and closed eyes prime across the blocks. Thus, in total, the experiment contained 96 trials for each prime × target condition. Each experimental trial consisted of the following events in sequence (Figure 1C): a fixation cross (500 ms), a gaze prime (100 ms), a blank screen (200 ms), and a word target presented until the participant’s response. Stimulus-onset-asynchrony (SOA) was 300 ms. The participants were seated 70 cm away from the screen and required to classify the target words as positive or negative, as quickly and accurately as possible, by pressing the “+” or “−” key (response key location was counterbalanced across participants). After the response, there was a 2000-ms interval before the next trial. Between the blocks, there was a 1-min break.

In the explicit rating task, the gaze pictures were shown to the participants one by one. The participants were required to evaluate the affective valence and arousal of each gaze stimulus on the 9-point Self-Assessment Manikin (SAM; Bradley and Lang, 1994) scales (1 = unpleasant/calm, 9 = pleasant/arousing). Each gaze stimulus was presented on the screen, first together with the arousal scale and then with the valence scale. Each face remained on the screen until the participant responded on both scales.

### Analyses of the Behavioral Data

Recognition accuracy and RTs were calculated from the priming task. Trials with incorrect responses to the word valence (5.54%) and trials with response latencies shorter than 2.5 standard deviations (SDs) below or above each participant’s mean (2.88%) were excluded. Log10-transform was applied to correct for non-normal distribution. A 3 (gaze: direct, averted, closed) × 2 (word valence: positive vs. negative) repeated measures analysis of variance (ANOVA) was performed on both recognition accuracy and RT data. For the rating task, one-way ANOVAs were conducted to test differences in the valence and arousal ratings for direct gaze, averted gaze and closed eyes. All statistical analyses were performed using the SPSS package. Huynh-Feldt correction was applied when appropriate. Bonferroni correction was performed for all multiple comparisons. For the sake of clarity, uncorrected degrees of freedom are reported.

### EEG Recording and Analyses

Electroencephalographic (EEG) activity was continuously recorded from 64 electrodes mounted in an electrode cap (actiCAP), amplified with a QuickAmp amplifier, and monitored with Brain Vision Recorder (Brain Products GmbH, Munich, Germany). The analog signal was amplified 26.55 times and the sampling rate was set to 1000 Hz. Using a common average reference, the EEG signals were referenced online to an average of all scalp electrodes. Additionally, vertical electro-oculogram (VEOG) was recorded with a pair of electrodes placed above and below the left eye. The FT9 electrode was used for recording horizontal electro-oculogram (HEOG). Electrode impedances were reduced under 30 kΩ.

Offline, the EEG signal was digitally filtered with a 0.5–30 Hz band-pass filter (24 dB/oct slope) and ocular-corrected using the Gratton/Coles algorithm (Gratton et al., 1983). Then, the corrected signal was segmented into 800-ms long epochs starting 100 ms prior to target onset. The mean voltage during the 100-ms pre-target period was used for baseline correction. Because we were mainly interested in ERPs after the target, using pre-target baseline, instead of pre-prime baseline, may be more appropriate as the pre-target ERPs may be shifted differently by different gaze primes (Hietanen and Astikainen, 2013). Artifacts were detected based on the following criteria: voltage step over 50 μV/ms, amplitude exceeding ±100 μV, and activity lower than 0.5 μV. Trials containing artifacts (8.0%) were rejected. Average waveforms in each experimental condition were calculated for each participant.

Selection of the electrode sites and time windows was based on visual inspection of the averaged waveforms and the findings from previous studies (Rossion et al., 2000; Kissler et al., 2009; Eder et al., 2011; Citron, 2012; Hietanen and Astikainen, 2013). For example, according to the previous literature, N170 and EPN have an occipitotemporal scalp distribution (Rossion et al., 2000; Citron, 2012), N400 is acquired from midline electrodes (Eder et al., 2011; Hietanen and Astikainen, 2013) and LPP peaks over the parietal lobe (Kissler et al., 2009). Therefore, the peak amplitude (an average of amplitudes within ±5 ms of the detected peak) and latency of P1, N170 and the mean amplitude of the EPN were determined from occipitotemporal channels P7 and P8, within a time window 80–160 ms, 160–260 ms and 300–400 ms, respectively. The mean amplitude of N400 was determined at midline electrodes Cz and CPz, within the time interval of 250–400 ms. Mean LPP amplitude was analyzed from channels CP1, CP2, P1, P2, CPz and Pz, within a time window of 400–700 ms. All ERP data were analyzed using repeated measures ANOVA with gaze and word valence as within-subject factors.

## Results

### Behavioral Data

**Figure 2:**
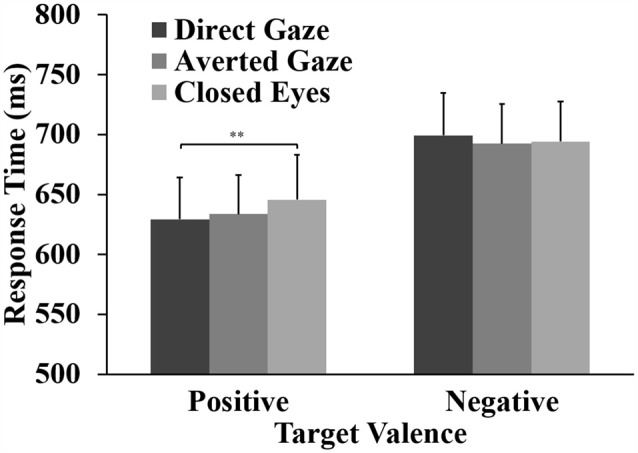
Means and standard errors for response times (RTs) to positive and negative targets preceded by direct gaze, averted gaze and closed eyes primes (**p < 0.01).

The ANOVA for the response accuracy data showed a significant main effect of word valence, *F*_(1,31)_ = 26.41, *p* < 0.001, $\eta_{\text{p}}^{2}$ = 0.460. Response accuracy for positive words (98%) was significantly higher than that for negative words (91%). For the RTs, the ANOVA showed a main effect of word valence, *F*_(1,31)_ = 46.84, *p* < 0.001, $\eta_{\text{p}}^{2}$ = 0.602. Participants responded faster to positive words (*M* = 636 ms) than to negative words (*M* = 695 ms). Importantly, the analyses showed the expected interaction between eye gaze and word valence, *F*_(2,62)_ = 5.36, *p* = 0.010, $\eta_{\text{p}}^{2}$ = 0.148. For positive words, a one-way ANOVA showed a main effect of gaze direction, *F*_(2,62)_ = 6.31, *p* = 0.003, $\eta_{\text{p}}^{2}$ = 0.169. Pairwise comparisons showed that responses to positive words were faster after direct gaze (*M* = 629 ms) than after closed eyes (*M* = 646 ms,* p* = 0.002, Cohen’s *d* = 0.69). The RT to positive words after averted gaze (*M* = 634 ms) did not differ significantly from the RTs to positive words preceded by direct gaze (*p* = 0.330, *d* = 0.29) and closed eyes (*p* = 0.265, *d* = 0.33). No effect of gaze direction was found on the RTs to negative words (*p* = 0.401; Figure 2).

### ERP Data

**Figure 3:**
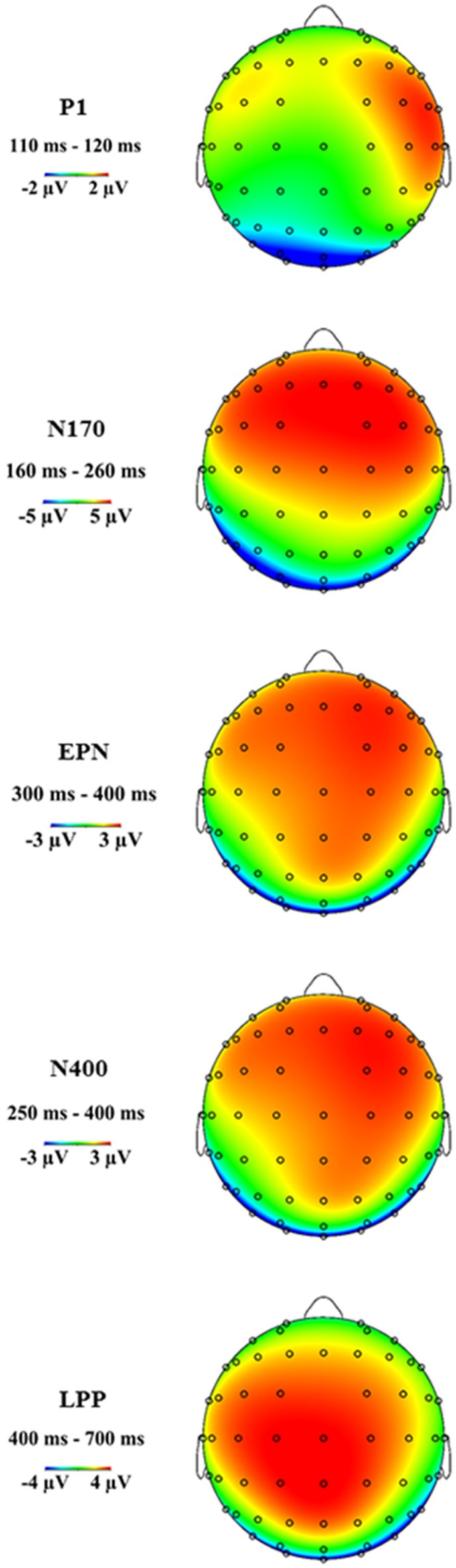
**Grand average event-related potential (ERP) topographies of the P1, N170, early posterior negativity (EPN), N400 and late positive potential (LPP) components at the indicated time windows**.

Figure 3 shows the grand average ERP topographies of the P1, N170, EPN, N400 and LPP components within the respective time windows.

### P1

**Figure 4:**
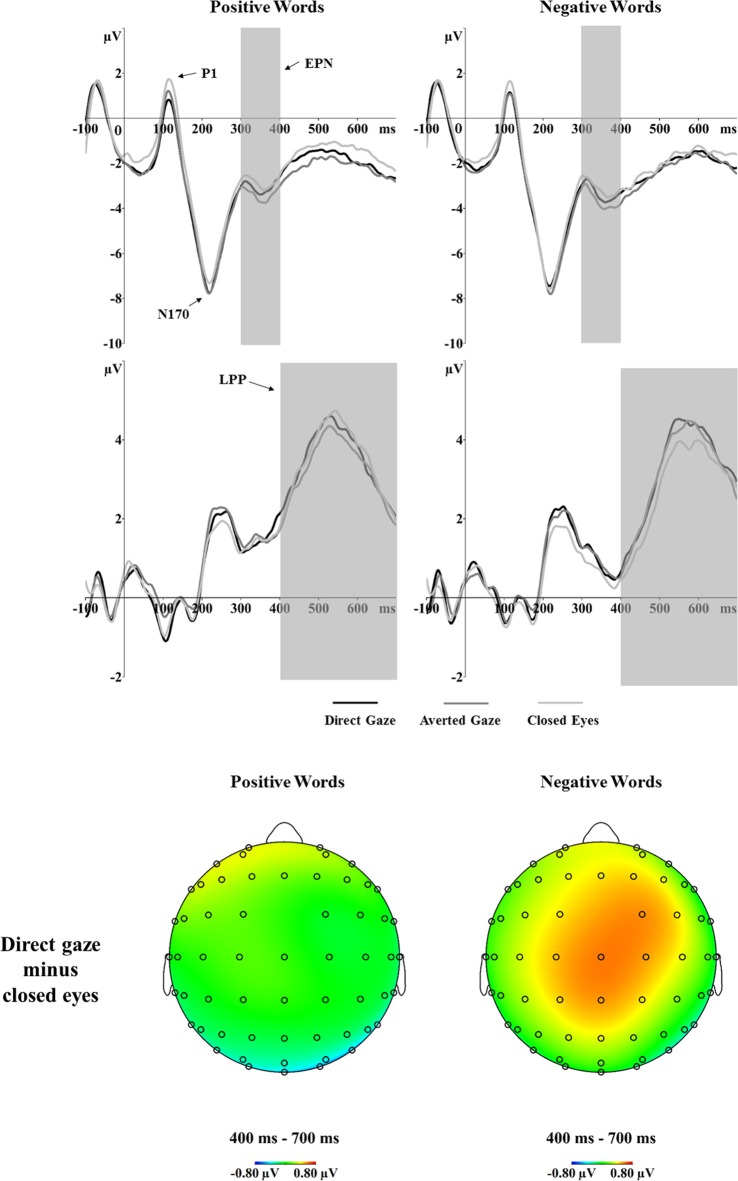
**Grand average ERPs of the P1, N170, EPN and LPP components to positive and negative words primed by direct gaze, averted gaze and closed eyes at the determined electrode sites (P7 and P8 for P1, N170 and EPN; CP1, CP2, P1, P2, CPz and Pz for LPP).** The voltage maps show the ERP difference (direct gaze minus closed eyes) for positive (left) and negative (right) words in the LPP time window.

For the P1 amplitudes, the main effect of gaze was significant, *F*_(2,56)_ = 15.97, *p* < 0.001, $\eta_{\text{p}}^{2}$ = 0.363. P1 amplitudes were larger after closed eyes (2.58 μV) than direct gaze (1.77 μV, *p* < 0.001, *d* = 0.92) and averted gaze (1.97 μV, *p* < 0.001, *d* = 0.90) primes (Figure 4). There was no significant main effect of word valence (*p* = 0.900) or an interaction between gaze and word valence (*p* = 0.454). For P1 latency, the analyses showed significant main effects of gaze, *F*_(2,56)_ = 4.73, *p* = 0.013, $\eta_{\text{p}}^{2}$ = 0.144. Specifically, the latency of P1 was shorter after averted gaze (118 ms) than closed eyes (122 ms, *p =* 0.017, *d* = 0.56) primes. The P1 latency after direct gaze (120 ms) did not differ from that after averted gaze or closed eyes (*p =* 0.197, *d* = 0.36; *p =* 0.854, *d* = 0.21, respectively). The interaction between gaze and word valence on P1 latency was not significant (*p =* 0.347).

### N170

The analyses of the N170 amplitudes revealed a significant interaction between gaze and word valence, *F*_(2,56)_ = 3.89, *p* = 0.026, $\eta_{\text{p}}^{2}$ = 0.122 (Figure 4). Separate one-way ANOVAs were conducted to investigate the effect of gaze primes on positive and negative words. For the positive words, a one-way ANOVA showed a main effect of gaze, *F*_(2,56)_ = 5.20, *p* = 0.008, $\eta_{\text{p}}^{2}$ = 0.157. Pairwise comparisons showed significantly larger N170 amplitudes in response to positive words after direct gaze (−8.68 μV) than after closed eyes primes (−8.01 μV, *p* = 0.005, *d* = 0.66). The N170 amplitudes for positive words preceded by averted gaze did not differ from those preceded by direct gaze (*p* = 1.000, *d* = 0.06) or by closed eyes (*p* = 0.111, *d* = 0.43). For negative words, there was no effect of gaze (*p* = 0.689). No significant effects were found in the analyses of N170 latency.

### EPN

The EPN analyses showed a main effect of gaze prime, *F*_(2,56)_ = 8.89, *p* < 0.001, $\eta_{\text{p}}^{2}$ = 0.241 (Figure 4). Overall, the EPN amplitude was larger (i.e., more negative) after averted gaze (−3.52 μV) than direct gaze (−3.18 μV, *p* = 0.008, *d* = 0.64) and closed eyes (−2.98 μV, *p* < 0.001, *d* = 0.73) primes. No other significant effects were found.

### N400

The analyses of the N400 mean activity showed that the main effects of gaze and word valence were significant. Overall, the N400 amplitude was smaller (i.e., more positive) after averted gaze (2.24 μV) than closed eyes (1.78 μV, *p* = 0.017, *d* = 0.60), while the N400 amplitudes after direct gaze (2.13 μV) did not differ from those after averted gaze and closed eyes (*p* = 0.940, *d* = 0.18; *p* = 0.154, *d* = 0.39, respectively), *F*_(2,56)_ = 5.31, *p* = 0.008, $\eta_{\text{p}}^{2}$ = 0.159. Finally, the N400 was smaller for positive (2.30 μV) than negative words (1.80 μV), *F*_(1,28)_ = 5.44, *p* = 0.027, $\eta_{\text{p}}^{2}$ = 0.163.

### LPP

The analyses showed a marginal main effect of word valence,* F*_(1,28)_ = 4.01, *p* = 0.055, $\eta_{\text{p}}^{2}$ = 0.125. Positive words (3.41 μV) elicited a larger LPP than did negative words (3.07 μV). Importantly, the analyses showed a significant interaction between gaze and word valence, *F*_(2,56)_ = 3.30, *p* = 0.044, $\eta_{\text{p}}^{2}$ = 0.105 (see Figure 4). Separate one-way ANOVAs for both positive and negative word conditions were not significant (*p* = 0.296; *p* = 0.117, respectively). However, as indicated by the ERP waveforms in Figure 4, the LPP amplitudes for negative words primed by different eye gaze conditions seemed to differ. Thus, we performed paired *t*-tests and the results suggested larger LPP responses to negative words preceded by direct gaze (3.23 μV) vs. closed eyes (2.83 μV, *p* = 0.033, *d* = 0.42).

### Explicit Ratings

**Figure 5:**
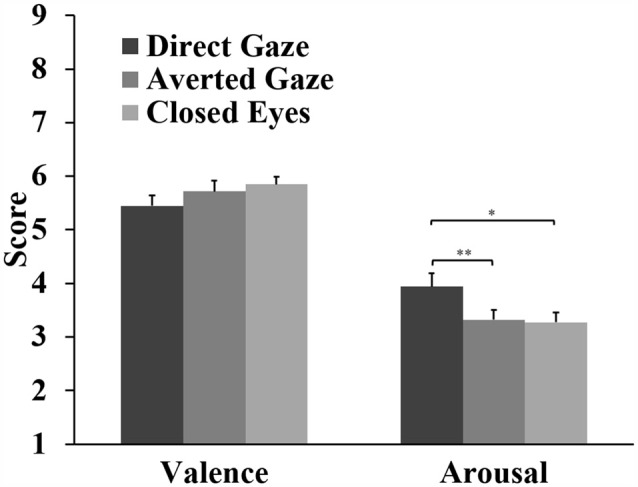
Means and standard errors for the valence and arousal ratings of the gaze stimuli (*p < 0.05; **p < 0.01).

For the valence ratings, the analyses showed a marginal main effect of gaze, *F*_(2,62)_ = 3.09, *p* = 0.053, $\eta_{\text{p}}^{2}$ = 0.091. Closed eyes (*M* = 5.85) and averted gaze (*M* = 5.72) were rated slightly more positive than direct gaze (*M* = 5.45), but these differences were not significant (*p* = 0.086, *d* = 0.42; *p* = 0.204, *d* = 0.34, respectively). There was a significant difference between gaze stimuli in arousal ratings, *F*_(2,62)_ = 7.22, *p* = 0.002, $\eta_{\text{p}}^{2}$ = 0.189. Direct gaze (*M* = 3.94) was rated more arousing than averted gaze (*M* = 3.32, *p* = 0.002, *d* = 0.70) and closed eyes (*M* = 3.27, *p* = 0.021, *d* = 0.52; Figure 5).

## Discussion

The present study employed affective priming and self-rating tasks to investigate implicit and explicit affective evaluation of eye gaze. ERPs in response to the target words were recorded during the affective priming task in order to examine the time course of the priming effects. The results of the affective priming task showed an interaction between eye gaze and word valence in the behavioral RT data, and revealed a priming effect for positive words, i.e., positive words were evaluated as being positive faster after direct gaze than after closed eyes. No priming effect was found for the negative words. The self-rating results showed that direct gaze was evaluated as less positive than closed eyes. The ERP analyses showed a clear priming effect on the N170 response and an interaction between eye gaze and word valence on the LPP response: a greater N170 amplitude for positive words after direct vs. closed eyes, and a slightly greater LPP for negative words after direct vs. closed eyes.

The behavioral results successfully replicate those of our previous study (Chen et al., 2016), and indicate that direct gaze implicitly elicits more positive affective reactions than closed eyes, whereas direct gaze is explicitly evaluated as less positive than closed eyes. Chen et al. (2016) suggested that because humans have a fundamental need to belong, and are motivated to form and maintain interpersonal relationships (Baumeister and Leary, 1995), direct gaze, as a signal of individuals’ approach tendency and intention of social communication, fulfils these needs and thus is automatically evaluated as a positive signal. However, when it comes to explicit evaluations, when individuals become aware of other people’s gaze and start to evaluate their corresponding feelings, their responses may become controlled and the initial and automatic responses are attenuated and suppressed. They may respond to the gaze stimuli in a reflective and analytic way, and may start to analyze the reason for the eye contact. Consequently, a direct gaze may evoke a feeling of uncertainty and, therefore, diminish positive feelings (Chen et al., 2016).

Importantly, the ERP results showed an interaction between eye gaze and word valence on the N170 and LPP responses. Specifically, a priming effect was found for positive words on the N170: responses for positive words were greater when preceded by direct gaze as compared to closed eyes. Previous research has shown greater N170 responses to targets preceded by affectively congruent compared to incongruent primes (Hietanen and Astikainen, 2013; Hinojosa et al., 2015). A plausible explanation is that the enhanced N170 in response to affectively congruent vs. incongruent pairs may reflect the integrative activation of affective valence by the contexts (primes) and the target stimuli, and the effect of this activation on visual processing (Hietanen and Astikainen, 2013; Diéguez-Risco et al., 2015). Thus, the N170 modulation indicates that direct gaze is affectively more congruent with positive words than closed eyes.

An interaction between eye gaze and word valence was also found on the LPP with slightly greater LPP for negative words preceded by direct gaze as compared to closed eyes. Prior studies have repeatedly reported that the LPP is modulated by affective congruence between the targets and preceding contexts, with affectively incongruent targets eliciting larger LPP amplitudes compared to affectively congruent targets (Werheid et al., 2005; Zhang et al., 2010; Herring et al., 2011; Hietanen and Astikainen, 2013). An enhanced LPP in response to affectively incongruent targets is interpreted as reflecting increased attentional resource allocation when the targets are unexpected or do not affectively match the preceding primes (Werheid et al., 2005; Zhang et al., 2010; Diéguez-Risco et al., 2015). Thus, the present LPP result indicates that direct gaze is affectively more incongruent with negative words compared with closed eyes. It should be noted, however, that the one-way ANOVAs for both positive and negative word conditions were not significant. This implies that the effects on the LPP were not robust.

According to the above interpretation of social needs being fulfilled by direct gaze, one may raise a question of why the effect of averted gaze did not significantly differ from direct gaze in both implicit affective evaluation and ERP measures, as averted gaze signals a tendency of avoidance. A possible explanation for this result might be the use of static gaze stimuli as primes, rather than dynamic gaze shifts or gaze of real persons. As compared with static gaze, dynamic and real gaze is more realistic and ecologically valid, and may lead to relatively stronger affective responses. Studies have shown that dynamic stimuli, relative to static stimuli, enhance neural responses and intensity judgments (Sato et al., 2004; Weyers et al., 2006). A series of studies by Hietanen et al. (2008) has demonstrated that gaze stimuli presented as pictures or by a real live person elicit differential behavioral and physiological responses (Pönkänen et al., 2011a,b). For example, Pönkänen et al. (2011a) reported greater N170 and EPN amplitudes to direct vs. averted gaze and closed eyes for the live gaze stimuli, but not for the gaze pictures. Similarly, a greater N170 to direct vs. averted gaze was reported in a study using dynamic gaze stimuli (Conty et al., 2007). Thus, we speculate that the employment of more realistic gaze stimuli may yield a clearer distinction between direct and averted gaze.

In the present study, the priming effect on N170 amplitudes was observed only with positive target words and the effect on LPPs only with negative target words. It is noteworthy that the priming effects shown on brain potentials are not always reported for both positive and negative targets. For example, Zhang et al. (2010) reported priming effects on LPP only for positive words; specifically, LPP for positive words was larger preceded by negative vs. positive pictures. They speculated that this was due to negative pictures generally eliciting stronger emotional reactions than positive pictures. This explanation can also be applied to the present results. As compared with closed eyes, the present as well as previous studies show that direct gaze is usually evaluated as emotionally more arousing and activating stronger autonomic responses (Helminen et al., 2011; Pönkänen et al., 2011a; Chen et al., 2016). As a result, direct gaze primes may lead to relatively stronger priming effects than closed eyes primes. Thus, for the N170 response, positive words presented in the context of direct gaze may elicit a stronger additive effect of integrative activation as compared with negative words presented in the context of closed eyes. With respect to the LPP, negative words presented in the context of direct gaze may elicit a stronger incongruency effect as compared with positive words presented in the context of closed eyes. It should be noted, however, that the observed interactions between the gaze stimuli and the positive and negative words cannot be explained by the differential arousal of direct gaze and closed eyes alone. We merely suggest that the automatic affective evaluations and the following affective priming effects may also depend on the affective arousal elicited by the primes.

For the behavioral results, we observed the priming effect with positive words but not with negative words. We have no obvious explanation for this finding. It should be noted that, in our previous study (Chen et al., 2016), the behavioral priming effects were observed for both positive and negative words. The present study had a similar experimental design as the previous study, with the exceptions that the present study included more target words and trials, only one prime—target SOA (instead of two), and the primes were supraliminally presented (without an additional subliminal condition). Nevertheless, we believe that this slight difference in the results shall not refute our conclusion.

The ERP analyses showed that the priming effects began to be reflected on brain activation as early as at the level of the N170 component. Prior studies investigating language comprehension have indicated that the influence of contextual information on lexical processing occurs approximately within the initial 200 ms following stimulus onset (Sereno et al., 2003). The present research accords with and extends the earlier findings by demonstrating that a gaze stimulus serving as an affective context also exerts an influence on affective word processing at an early stage of neural processing.

The present study showed that the P1, EPN and N400 components were not modulated by the affective congruence between primes and targets. A few prior affective priming studies have reported priming effects on the P1 and EPN (Hietanen and Astikainen, 2013; Rampone et al., 2014; Sianipar et al., 2015). The reasons for the discrepant findings may be related to differences in the methods between the studies. For example, the target stimuli used by Hietanen and Astikainen (2013) were facial expression pictures, whereas visually presented words were used in the present study. As compared with affective faces, the affective meaning of words is ontogenetically learned and symbolic and, therefore, the processing of affective information from words and faces may be different (Schacht and Sommer, 2009). Several affective priming studies with words as targets have reported a larger N400 in response to affectively incongruent targets (Zhang et al., 2006, 2010; Eder et al., 2011). However, some studies have not observed an N400 modulation (Herring et al., 2011; Kissler and Koessler, 2011), or have even reported reversed affective priming effects (Aguado et al., 2013; Hietanen and Astikainen, 2013). Herring et al. (2011) conducted three affective priming experiments with either affective picture or word pairs and reported a priming effect on the LPP, but not on the N400. They argued that these results may suggest a dissociation in the processes of semantic and affective priming: the LPP is modulated by affective mismatch, whereas the N400 may largely depend on the semantic connectedness between the primes and targets.

A limitation that should be addressed in future research is related to visual low-level differences between different gaze stimuli. For example, direct gaze has a higher sclera-iris contrast than closed eyes, and is geometrically more symmetric than averted gaze. One may speculate that the effects reported in our studies were driven by low-level features of the eyes when presented briefly, rather than by social evaluation as we suggested. To examine this, future studies could include face stimuli with frontal and rotated head orientations, and appropriate control stimuli, such as scrambled faces with direct gaze, averted gaze and closed eyes.

## Conclusion

Corresponding to our hypotheses, the present study found that the behavioral data successfully replicated previous findings (Chen et al., 2016), indicating that direct gaze is implicitly evaluated as more positive than closed eyes, whereas it is evaluated as less positive in explicit evaluations. The ERP analyses showed a priming effect on the N170 and an interaction on LPP responses: a larger N170 for positive words after direct vs. closed eyes and a slightly larger LPP for negative words after direct vs. closed eyes. These results indicate that direct gaze, as compared with closed eyes, is perceived as more affectively congruent with positive words, and as more affectively incongruent with negative words. The observed priming effect on the N170 also indicates that gaze stimuli influence the subsequent affective word processing at an early stage of information processing.

## Author Contributions

All the four authors were involved in the experimental design, data collection and analysis. TC, MJP and JKH were involved in manuscript writing and data interpretation work.

## Funding

This work was supported by the GATE Erasmus Mundus Project (to TC) and the Academy of Finland (grant #275519 to MJP; MIND program grant #266187 to JKH).

## Conflict of Interest Statement

The authors declare that the research was conducted in the absence of any commercial or financial relationships that could be construed as a potential conflict of interest.

## Footnotes

^1^http://www.daz3d.com/
